# Nanoengineered chiral Pt-Ir alloys for high-performance enantioselective electrosynthesis

**DOI:** 10.1038/s41467-021-21603-8

**Published:** 2021-02-26

**Authors:** Sopon Butcha, Sunpet Assavapanumat, Somlak Ittisanronnachai, Veronique Lapeyre, Chularat Wattanakit, Alexander Kuhn

**Affiliations:** 1 University of Bordeaux, CNRS UMR 5255, Bordeaux INP, Site ENSCBP, 33607 Pessac, France; 2 School of Molecular Science and Engineering and School of Energy Science and Engineering, Vidyasirimedhi Institute of Science and Technology, 21210 Rayong, Thailand

**Keywords:** Synthetic chemistry methodology, Electrocatalysis, Porous materials

## Abstract

The design of efficient chiral catalysts is of crucial importance since it allows generating enantiomerically pure compounds. Tremendous efforts have been made over the past decades regarding the development of materials with enantioselective properties for various potential applications ranging from sensing to catalysis and separation. Recently, chiral features have been generated in mesoporous metals. Although these monometallic matrices show interesting enantioselectivity, they suffer from rather low stability, constituting an important roadblock for applications. Here, a straightforward strategy to circumvent this limitation by using nanostructured platinum-iridium alloys is presented. These materials can be successfully encoded with chiral information by co-electrodeposition from Pt and Ir salts in the simultaneous presence of a chiral compound and a lyotropic liquid crystal as asymmetric template and mesoporogen, respectively. The alloys enable a remarkable discrimination between chiral compounds and greatly improved enantioselectivity when used for asymmetric electrosynthesis (>95 %ee), combined with high electrochemical stability.

## Abstract (web summary)

Tremendous efforts have been made over the past decades regarding the development of materials with enantioselective properties. Here, the authors present a nanostructured chiral imprinted metal alloy that exhibits high enantioselectivity and stability for the electrosynthesis of chiral molecules.

## Introduction

Homochirality is naturally present, especially, in organisms among others as amino acids (left-handed) and sugars (right-handed)^1^. A chiral molecule exits in two enantiomeric forms, which are mirror images of each other, but are non-superposable stereoisomers. In a biological context, it is important to note that only one enantiomer tends to be active, whereas the other one is inactive or even toxic^2^. Therefore, the development of chiral technologies to obtain enantiomerically pure compounds (EPCs) is of crucial importance, due to the large variety of potential applications in fields ranging from pharmaceutics to food industry^3–6^. Up to date, there are various technologies allowing the production of EPCs^5,6^. Enantioselective synthesis is considered as one of the most interesting approaches, compared to the separation of racemic mixtures, due to its high product specificity and less waste formation^7,8^. Homogeneous catalysis, based on coordination complexes, as well as enzymatic catalysis have shown high efficiency under mild conditions over the past decades^3,7,9^. However, heterogeneous catalysis can have additional advantages in terms of an easier regeneration and improved catalytic properties, allowing a broadening of the substrate spectrum for the production of chiral molecules^10^.

Metals are considered as promising materials for heterogeneous chiral catalysis^11,12^ due to their rigid structure, combined with a high conductivity, versatile surface modification strategies, and mechanical or chemical stability, often limited in the case of other materials^13–15^. Presently, there are several possible approaches with respect to the generation of chiral features in or on metals: (i) molecular adsorption or grafting;^16–18^ (ii) breaking the symmetry of bulk metal crystals^19,20^; and (iii) chiral molecular imprinting inside a metal matrix^21–28^. Among these approaches, molecular imprinting of metals has recently attracted attention due to the simple preparation process and the easy preservation of molecular information^15,29^. An early example of generating chiral features in metal derivatives has been the imprinting CuO films, obtained by electrodeposition in the presence of tartrate enantiomers. Noticeably, they displayed different electrooxidation rates for (R,R)- and (S,S)-tartrate as a function of the imprinted chiral configuration^21^. Apart from electrodeposition, chiral-imprinted metallo-organic hybrid materials based on Pd modified with cinchona alkaloids were also synthesized by a chemical method^22^. The resulting Pd nanoparticles retain a specific chiral character even after removal of the template molecule.

Recently, we have demonstrated the possibility to generate a new type of chiral metals, namely chiral-imprinted mesoporous metals (CIMMs), using platinum^23–26^ and nickel^27^. The strategy is based on the synergy of molecular imprinting and the electrodeposition of mesoporous metal structures^30^. These advanced materials exhibit interesting chiral features, combined with improved mass transport due to the presence of mesopores, and can be used for a wide range of applications, from enantioselective analysis^23,24^ and chiral separation^31^ to chiral actuators^32^ and asymmetric synthesis^25–27^. In the latter case, chiral-encoded mesoporous metals were employed as electrodes for the electroreduction of prochiral molecules like phenyl glyoxylic acid and acetophenone in order to obtain (R)- or (S)-mandelic acid (MA)^25^ and (R)- or (S)-phenylethanol (PE)^26,27^, respectively. Initially, only a rather modest enantiomeric excess (%ee) has been obtained, however, using pulsed electroreduction resulted in a greatly improved enantiomeric excess^26,27^. Despite this enantioselectivity, the synthesis strategy is rather time-consuming and, most importantly, the stability of the chiral features is limited, thus preventing practical applications^26,27^. Consequently, the development of chiral metal matrices, which not only allow highly enantioselective synthesis, but are also able to retain their chiral character for extended periods of time is a crucial requirement for enabling real-world applications.

In the present work, we address this key challenge by developing a nanostructured chiral-imprinted metal alloy that not only exhibits very pronounced enantioselectivity, but also has a significantly increased stability when used as an electrode material for the synthesis of chiral molecules.

## Results

### Preparation of chiral-imprinted mesoporous Pt-Ir alloys

For improving the structural stability of chiral-imprinted metals, i.e., avoiding a gradual loss of the chiral information, the design of metal alloys is a promising strategy^33^. The usually higher stability of metal alloys can be attributed to the different size of the metal atoms, eventually retarding dislocation in the bulk structure^34^. Compared to monometallic Pt, Pt alloys generally exhibit greatly enhanced hardness and strength. Typically, various metals, such as ruthenium (Ru), rhodium (Rh), iridium (Ir), and gold (Au) have been used to form alloys with Pt in order to improve its mechanical properties. Among them, Ir is one of the most excellent promoters to increase the hardness, up to four times higher than that of the pure Pt, for example, when using Pt-Ir alloys with 5–30 wt% of Ir^35,36^. Furthermore, Pt-Ir alloys show improved (electro-)catalytic activity due to compressive surface strain induced by the lattice mismatch between Pt and Ir, which alters the surface energy^12,37,38^. The electrochemical stability is also enhanced, because Ir can tolerate rather harsh electrochemical conditions as has been demonstrated, for example, in the context of alcohol oxidation^39^ and oxygen reduction^37,38^. In addition, Ir is easily incorporated in the alloy as it has the same lattice structure as Pt. Moreover, Ir salts can be electroreduced by applying potentials ranging from 0.0 to −0.1 V vs. Ag/AgCl, similar to the ones needed for Pt salt reduction^36,40^. This illustrates the benefits of incorporating Ir into bulk Pt, especially when following an electrodeposition route.

**Fig. 1:**
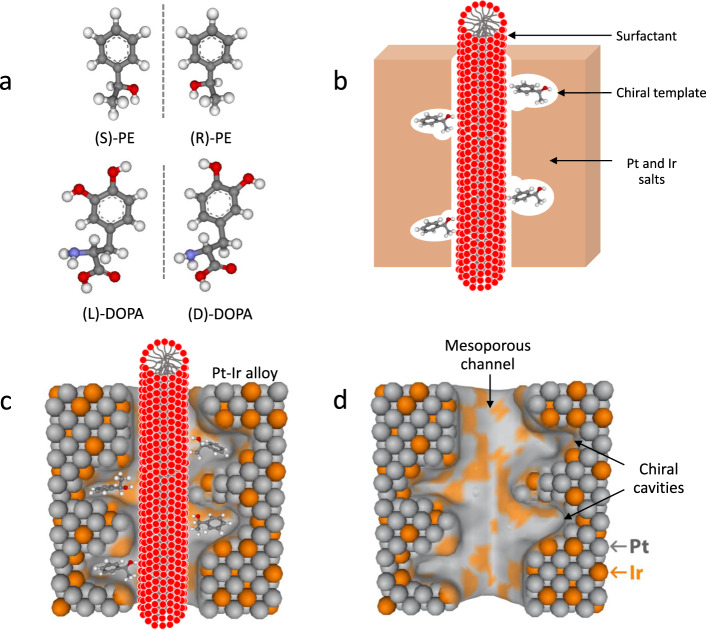
Synthesis of chiral-imprinted mesoporous Pt-Ir alloys. **a** Molecular structure of (R)-PE, (S)-PE, (L)-DOPA, and (D)-DOPA. **b** Formation of columns of non-ionic surfactant decorated with chiral template in the presence of a mixture of Pt and Ir salts. **c** Growth of alloyed Pt and Ir around the supramolecular surfactant structure and chiral templates by co-electrodeposition. **d** Final chiral-imprinted mesoporous Pt-Ir alloy after removal of the templates.

Currently, there are several preparation methods for bimetallic mesoporous Pt alloys. One strategy is based on the use of a lyotropic liquid crystal (LLC) as mesoporogen, and porous Pt-Au, as well as Pt-Ru could be obtained by co-electrodeposition^41^. However, so far the electrogeneration of mesoporous chiral Pt-Ir has not been explored, and therefore we have chosen Ir as a promoter for the preparation of Pt-Ir in order to obtain ultra-stable chiral-encoded mesoporous Pt-Ir alloys. The mesoporous Pt-Ir alloys have been successfully synthesized by co-electrodeposition of Pt and Ir salts from mixtures with a 85:15 at.% ratio (a weight ratio of [PtCl_6_^2−^]/[IrCl_6_^2−^] = 4.5), in the simultaneous presence of chiral templates and surfactant to form a LLC phase on a gold-coated glass slide, acting as a substrate. Figure 1a illustrates the structures of the chiral templates (R)-PE, (S)-PE, (L)-DOPA, and (D)-DOPA, which have been chosen as model compounds in this work. They adsorb at the external surface of the non-ionic surfactant (polyethylene glycol hexadecyl ether, Brij^®^ C10), forming columns with a self-assembled hexagonal arrangement (H_1_) (Fig. 1b). Subsequently, electrodeposition is carried out to deposit Pt and Ir around the supramolecular template structure (Fig. 1c). Finally, after removal of the templates, specific chiral sites at the internal walls of the mesopores are obtained (Fig. 1d).

**Fig. 2:**
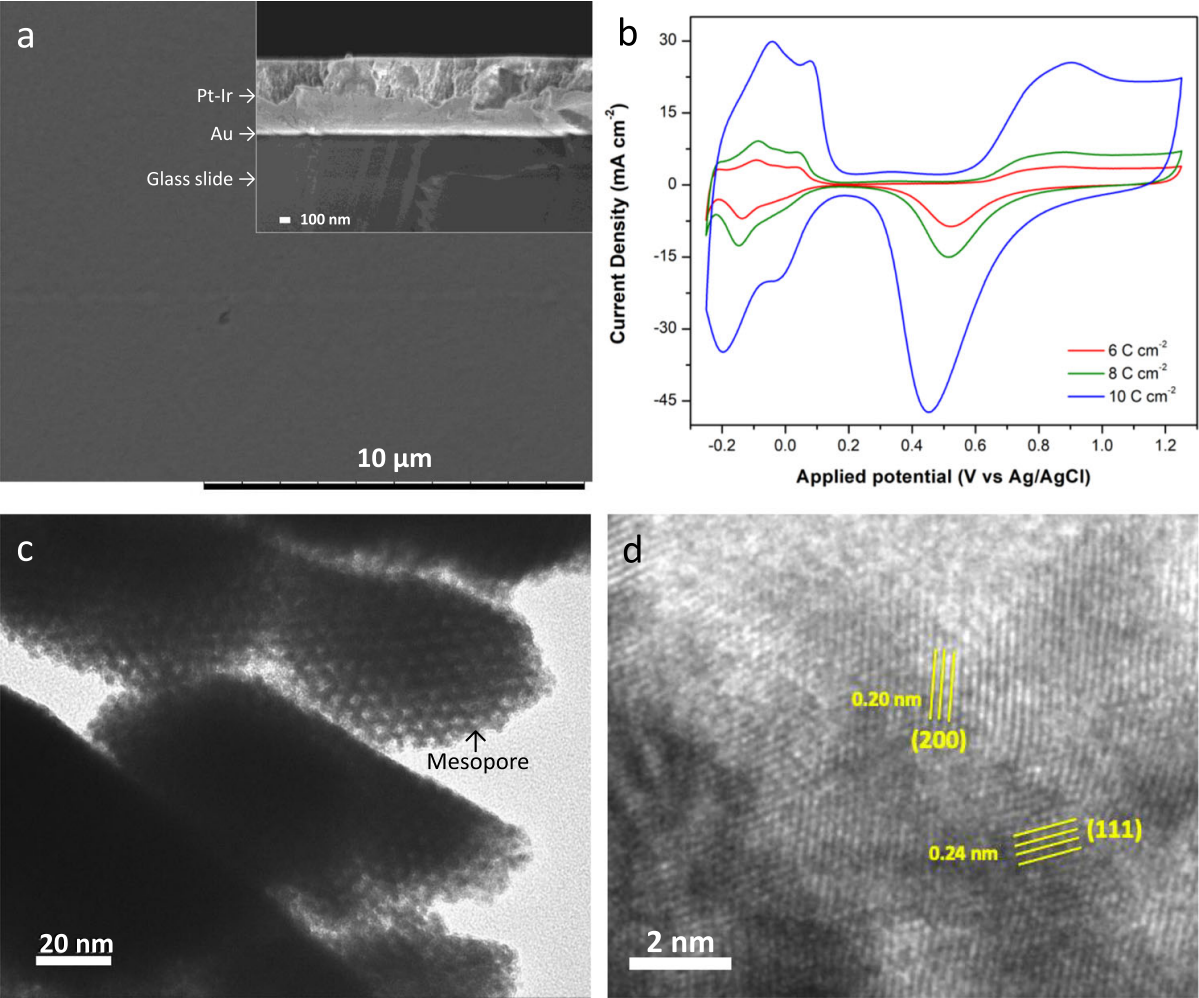
Characterization of the mesoporous Pt-Ir electrodes. **a** SEM image (top view) of mesoporous Pt-Ir electrodeposited at −0.05 V with a deposition charge density of 8 C cm^−2^ (scale bar 10 µm). The inset shows a cross-sectional SEM image of a mesoporous Pt-Ir film obtained with a deposition charge density of 8 C cm^−2^ (scale bar of 100 nm). **b** Cyclic voltammograms in 0.5 M H_2_SO_4_ at a scan rate of 100 mV s^−1^of mesoporous Pt-Ir electrodes, obtained by varying the deposition charge densities. **c** TEM image of an ultra-thin Pt-Ir layer revealing the well-organized mesopores (scale bar of 20 nm). **d** HR-TEM image of an ultra-thin Pt-Ir alloy layer (scale bar of 2 nm).

However, rates of electrodeposition can be different for Pt and Ir. Therefore, in order to ensure a homogeneous incorporation of Ir into the Pt bulk, the effect of electrodeposition potential on the film formation and composition was investigated using H_2_PtCl_6_ and H_2_IrCl_6_ as metal precursors, with a deposition charge density of 10 C cm^−2^. Rough surfaces are observed when applying a potential of −0.20 V vs. Ag/AgCl, as can be seen in the scanning electron microscopy (SEM) images (Supplementary Fig. 1a). In strong contrast to this, a very homogeneous smooth surface is obtained for an applied potential of −0.10 and −0.05 V (Supplementary Fig. 1b and Fig. 2a, respectively). These observations are consistent with the fact that the electrochemically active surface area (ECSA), calculated by integrating the peak regions of hydrogen adsorption/desorption obtained by cyclic voltammetry (CV) in H_2_SO_4_ solution^23,26,42,43^ (Supplementary Fig. 1c), is higher for the deposit generated with the more negative potential, due to the very rough external surface (Supplementary Table 1). However, there is no detectable Ir content in the case of the more negative deposition potentials (−0.1 and −0.2 V). According to the literature, the electrodeposition rate and charge efficiency for Ir depends on the applied potential in the range from −0.1 to 0.0 V vs. Ag/AgCl, whereas for Pt, charge efficiency starts to remain roughly constant at −0.05 V vs. Ag/AgCl^40^. It is therefore reasonable to assume that a deposition potential of −0.05 V should be suitable for the synthesis of a Pt-Ir alloy containing a small amount of Ir, as revealed by energy dispersive X-ray spectrometry (EDS) (Supplementary Table 1).

Apart from fine-tuning the deposition potential, the deposit thickness is another important parameter having an impact on the film composition and properties. This can be easily varied by changing the deposition charge density. Layers with different thickness could be obtained by varying the charge density from 6 to 10 C cm^−2^. As expected, the CVs of mesoporous Pt-Ir electrodes with varying thickness (Fig. 2b) show features similar to mesoporous Pt (Supplementary Fig. 1d)^23,26^. The presence of a large amount of Pt precursor compared to Ir in the initial LLC gel, combined with the lower electrodeposition rate of Ir, results in a preferential deposition of Pt. Most importantly, the active surface area gradually increases with increasing deposition charge density; however, the Ir content significantly decreases (Supplementary Table 1). The reason for this apparent decrease in Ir content relates to the fact that EDS is sensing the outermost surface, whereas the Pt-Ir alloy is preferably formed in the lower part of the chiral-imprinted Pt-Ir film. The latter effect can be ascribed to a gradual depletion of Ir salt in the solution in front of the growing interface, due to its rather already small initial concentration. Therefore, when higher deposition charge densities are used, the deposit contains higher percentages of Ir in the initial stages of growth, but later, less and less Ir is incorporated into the growing layer. Therefore, the deposition charge density used in this work did not exceed 8 C cm^−2^, in order to obtain an acceptable Ir content. A cross-sectional SEM image (inset in Fig. 2a) illustrates the uniform thickness (approximately 900 nm) of the Pt-Ir film over the entire area, implying that the bimetallic film is successfully obtained when performing the electrodeposition at −0.05 V and with 8 C cm^−2^. In order to further confirm the mesoporous structure of the prepared Pt-Ir film, transmission electron microscopy (TEM) measurements are performed with a thin piece of the deposit. A well-ordered hexagonal mesoporous structure with pore dimensions of around 5 nm is clearly visible in Fig. 2c, which is in agreement with the theoretically expected pore size for this type of surfactant^30^.

In order to confirm the alloy structure of the Pt-Ir electrodes, two representative samples of thinner and thicker films, namely Pt-Ir 4 C and Pt-Ir 8 C, were prepared by controlling the deposition charge density during co-electrodeposition. X-ray diffraction patterns (XRD) of both Pt-Ir electrodes show intense diffraction peaks at 2θ of 40.0 and 46.4 degree corresponding to the plane indices of (111) and (200), respectively, which are shifted to higher degrees compared to the standard card of Pt (Supplementary Fig. 2). These observations already seem to indicate that Pt and Ir are forming an alloy with a face-center cubic (fcc)^39,44^. This is also confirmed by high-resolution transmission electron microscopy (HR-TEM) images of the ultra-thin Pt-Ir sample (Fig. 2d). The (111) and (200) lattice fringes are observed with *d*-spacing values of 0.24 and 0.20 nm, respectively. The lattice constants of Pt-Ir 4 C and Pt-Ir 8 C are not significantly different, but obviously smaller and higher than those of Pt and Ir, respectively (Supplementary Fig. 2b). This confirms that Pt and Ir are alloyed, inducing a compressive strain, which might influence the electrochemical activity^37^.

**Fig. 3:**
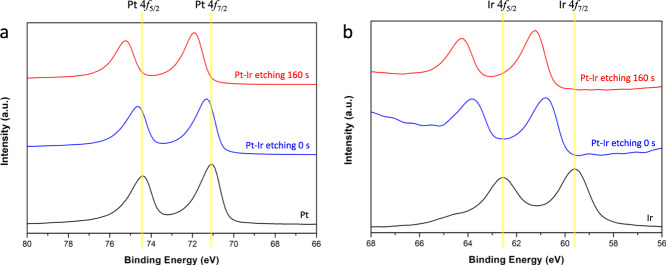
XPS spectra of the electrodes. **a**, **b** Narrow-scan XPS spectra of Pt and Ir in the binding energy (BE) regions of Pt and Ir, respectively, compared to those of a fresh Pt-Ir 8 C alloy sample at different etching times of 0 and 160 s.

The composition of the prepared Pt-Ir films was further investigated by X-ray photoelectron spectroscopy (XPS). As shown in Fig. 3a, b, standard monometallic Pt and Ir (black) exhibit main characteristic peaks at 71.0 and 74.4 eV for Pt 4*f*_7/2_ and Pt 4*f*_5/2_, as well as at 59.7 and 62.7 eV for Ir 4*f*_7/2_ and 4*f*_5/2_, respectively^42,45^. Interestingly, the characteristic doublet peaks of either Pt or Ir are significantly shifted to higher binding energies for the Pt-Ir films in the case of Pt-Ir (0.3 and 1.1 eV as shown in blue for Pt and Ir, respectively), and there is no significant difference between Pt-Ir 4 C and Pt-Ir 8 C (Supplementary Fig. 3a, b). In order to further examine the alloy structures, XPS depth-profile analysis was performed by bombarding Pt-Ir surfaces with an argon ion gun. Supplementary Fig. 3c displays the evolution of the spectra of Pt-Ir 8 C in the binding energy (BE) range between 90 and 55 eV, as a function of increasing etching time. When etching for 160 s, a significant shift of the double peaks of Pt and Ir to higher BE values was observed (Fig. 3a, b (red)). The reason of this shift is a charge transfer between Pt and Ir^42^. It is therefore reasonable to assume that Pt and Ir are forming an alloy structure. Moreover, the exact amount of Pt and Ir in the Pt-Ir alloy could be measured by elemental analysis with inductively coupled plasma optical emission spectroscopy (ICP-OES), resulting in approximately 90 and 10 at.% of Pt and Ir, respectively (Supplementary Table 2).

### Enantioselective recognition properties

**Fig. 4:**
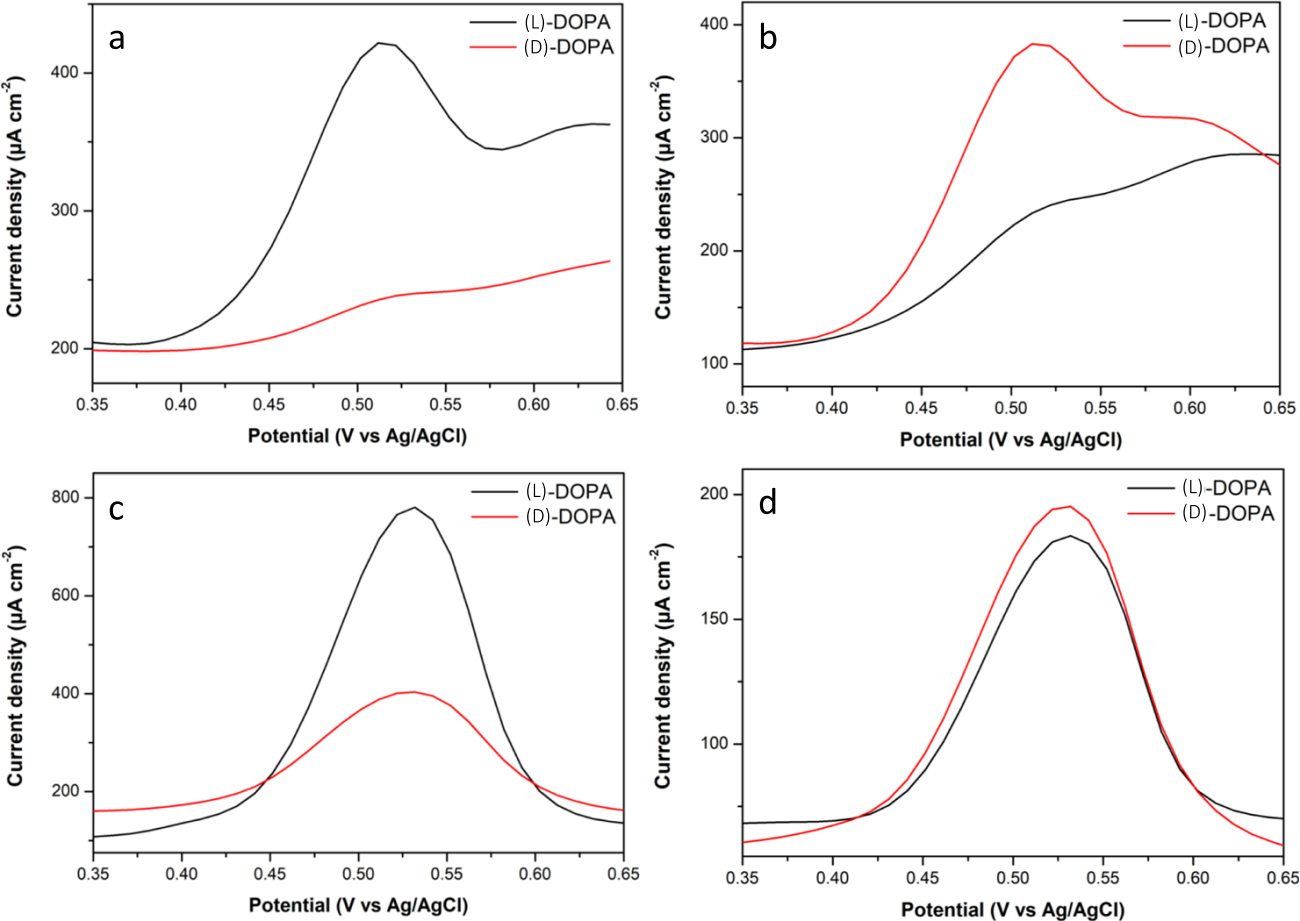
Enantioselective detection. Differential pulsed voltammograms (DPVs) of (L)-DOPA (black) and (D)-DOPA (red) electrooxidation recorded with different Pt-Ir electrodes: **a**, **b** Signals recorded for fresh (L)-DOPA and (D)-DOPA-imprinted mesoporous Pt-Ir alloys, respectively. DOPA electrooxidation on (L)-DOPA-imprinted mesoporous Pt-Ir alloys after trying to erase the chiral information by cycling the potential in 0.5 M H_2_SO_4_ for: **c** 10 cycles in the range from −0.20 to 1.20 V vs. Ag/AgCl, and **d** 40 cycles from −0.20 to 1.80 V vs. Ag/AgCl. All experiments were performed in 4 mM (L)- or (D)-DOPA in 50 mM HCl solution as supporting electrolyte (pH = 1.4) with electrodes synthesized with a deposition charge density of 6 C cm^−2^.

The chiral discrimination abilities of imprinted Pt-Ir films were tested by electrooxidation of (D)-DOPA and (L)-DOPA using differential pulsed voltammetry (DPV). A potential region from 0.35 to 0.65 V was chosen, because it allows DOPA oxidation without interference with Pt electrooxidation. As expected, in the case of non-imprinted mesoporous Pt-Ir, there is no discrimination between (D)- and (L)-DOPA (Supplementary Fig. 4a). On the other hand, a (L)-DOPA-imprinted Pt-Ir film shows a much higher current density for (L)-DOPA compared to the opposite enantiomer (Fig. 4a). In strong contrast to this, for the (D)-DOPA-imprinted Pt-Ir alloy, the electrode preferentially converts the (D)-DOPA enantiomer, as illustrated again by a higher oxidation peak (Fig. 4b). These observations confirm that chiral information can be retained by the mesoporous Pt-Ir surfaces, even after complete removal of the chiral templates. Compared with chiral-imprinted monometallic Pt (Supplementary Fig. 4b), a remarkably higher enantioselective recognition ability is observed for imprinted Pt-Ir. The degree of recognition efficiency can be characterized more quantitatively by considering the integrated peak areas of current density in the potential region of DOPA oxidation (Supplementary Table 3). Relative oxidation peak areas of (L)-/(D)-DOPA or (D)-/(L)-DOPA are reported as A_L_/A_D_ or A_D_/A_L_, respectively. These values follow a systematic trend. Similar values of A_L_/A_D_ and A_D_/A_L_ are obtained for fresh bimetallic Pt-Ir electrodes encoded with (L)-DOPA and (D)-DOPA (14.5 and 13.5, respectively); on the other hand, the A_L_/A_D_ ratio for (L)-DOPA-imprinted monometallic Pt displays only 1.5. This clearly demonstrates the greatly improved enantioselective recognition ability of Pt-Ir alloys. The enhanced recognition activity of chiral-imprinted Pt-Ir might have different reasons: (i) a limited adsorption of the chiral molecules on unselective sites due to the smoother external surface of porous Pt-Ir compared to porous monometallic Pt which can exhibit Pt islands where unspecific adsorption can occur^46^ (SEM images of Supplementary Fig. 5a, b); (ii) the modification of adsorption properties related to the atomic arrangement (decrease in the Pt–Pt bond distance) and a variation of the electronic structure of platinum metal;^47,48^ (iii) the formation of a more asymmetric environment due to the lower symmetry of the metal structures resulting from the introduction of a different type of metal atoms in the bulk phase^33,49^.

The electrochemical stability of the chiral-imprinted alloys was further studied in additional experiments by oxidizing the imprinted metal matrix on purpose at quite high positive potentials (>0.65 V vs. Ag/AgCl). Pure Pt typically starts to get oxidized at these potential values, accompanied by a reorganization of the surface atoms, eventually resulting in a destruction of the chiral information. As expected, the electrooxidation current of (L)- and (D)-DOPA at monometallic Pt electrodes encoded with (L)-DOPA is identical (A_L_/A_D_ close to 1.0) after 10 cycles from −0.20 to 1.20 V in H_2_SO_4_ (Supplementary Fig. 6a). However, in big contrast to that, a remarkable discrimination of (L)- and (D)-DOPA is preserved for (L)- and (D)-DOPA-imprinted mesoporous Pt-Ir electrodes, even after 10 full cycles in H_2_SO_4_ (Fig. 4c and Supplementary Fig. 6b). Significant enantiorecognition properties of Pt-Ir can be still maintained even after trying to continuously erase the chiral information for 20 cycles (Supplementary Fig. 7a, b) and 30 cycles in the potential range from −0.20 to 1.40 V (Supplementary Fig. 7c, d). Nearly constant A_L_/A_D_ or A_D_/A_L_ values of around 1.3 are obtained (Supplementary Table 3). In order to confirm that such an unusual electrochemical stability is not due to some experimental artifacts, even more extreme conditions were used to destroy the chiral information. Oxidation of the electrodes in acid solution was carried out in an extended potential range from −0.2 to 1.8 V for 40 cycles. As can be seen in Fig. 4d and Supplementary Fig. 7e for (L)- and (D)-DOPA-encoded Pt-Ir, these very aggressive conditions finally lead to a complete loss of chiral information with A_L_/A_D_ or A_D_/A_L_ values close to 1.0. All these observations confirm that imprinted mesoporous Pt-Ir electrodes display an exceptional electrochemical stability with respect to a loss of chiral information, and such a performance cannot be achieved with monometallic Pt.

### Enantioselective electrosynthesis

Chiral-imprinted metal electrodes can be used not only for enantioselective recognition, but also for generating a high %ee during asymmetric electrosynthesis, as has been reported previously for monometallic electrodes^26,27^. However, the long global synthesis time, combined with a rather limited stability of the chiral information in monometallic Pt and Ni, is a major roadblock for practical applications. In order to circumvent these limitations, we explored the use of the designed highly stable Pt-Ir alloys (vide supra) for the asymmetric electrosynthesis of chiral compounds. This is an interesting challenge, since the efficiency of chiral electrosynthesis might be greatly improved in terms of the activity and stability when using alloys instead of pure metal.

In order to study these potentially very beneficial effects, the electroreduction of ketone functions (C = O) to alcohol (–OH) has been chosen as a model reaction. Acetophenone is a suitable candidate because it can be reduced to 1-phenylethanol (1-PE). A second product of the reaction might be 2,3-diphenyl-2,3-butanediol^50^, however by optimizing the experimental conditions, such as the pH of the supporting electrolyte, a rather selective production of PE can be achieved.

(S)-PE was used as a chiral template with a weight ratio of 0.15 for PE/(PtCl_6_^2−^ + IrCl_6_^2−^) analog to the conditions used in previous reports^26^. The reaction products were analyzed by high-performance liquid chromatography (HPLC) using a chiral column. The chromatograms of acetophenone, (R)-PE and (S)-PE, are clearly separated with retention times of 10.5, 13.0, and 13.9 min, respectively (Supplementary Fig. 8). The electrosynthesis was carried out just at the onset potential of acetophenone reduction at −0.4 V in 1 M NH_4_Cl as a supporting electrolyte and an initial solution pH of 5.0 in order to prevent proton reduction at more negative potentials (Supplementary Fig. 9). As mentioned above, the reaction pathway can be shifted to a preferential production of 1-PE by lowering the solution pH^50^. Therefore, reaction mixtures with a different pH in the range from 5.0 to 2.0, adjusted by 1 M HCl, were tested. The applied potentials were increased by approximately +60 mV per pH unit, following the theoretical relationship between potential and pH derived from Nernst equation^51^. The results, summarized in Supplementary Table 4, reveal that when decreasing the pH from 5.0 to 4.0, the %ee of PE increases remarkably from 28% to 49%. However, the enantiomeric excess is dramatically decreased when the solution pH is even lower (24% and 2% at pH 3.0 and 2.0, respectively). This might be related to the fact that the competing reaction to form a racemic mixture becomes more favorable when an excessive number of adsorbed protons is available, facilitating the unspecific protonation of the intermediate species. Therefore, the most appropriate condition for chiral electrosynthesis is an applied potential of −0.35 V in a solution of pH 4.0.

**Fig. 5:**
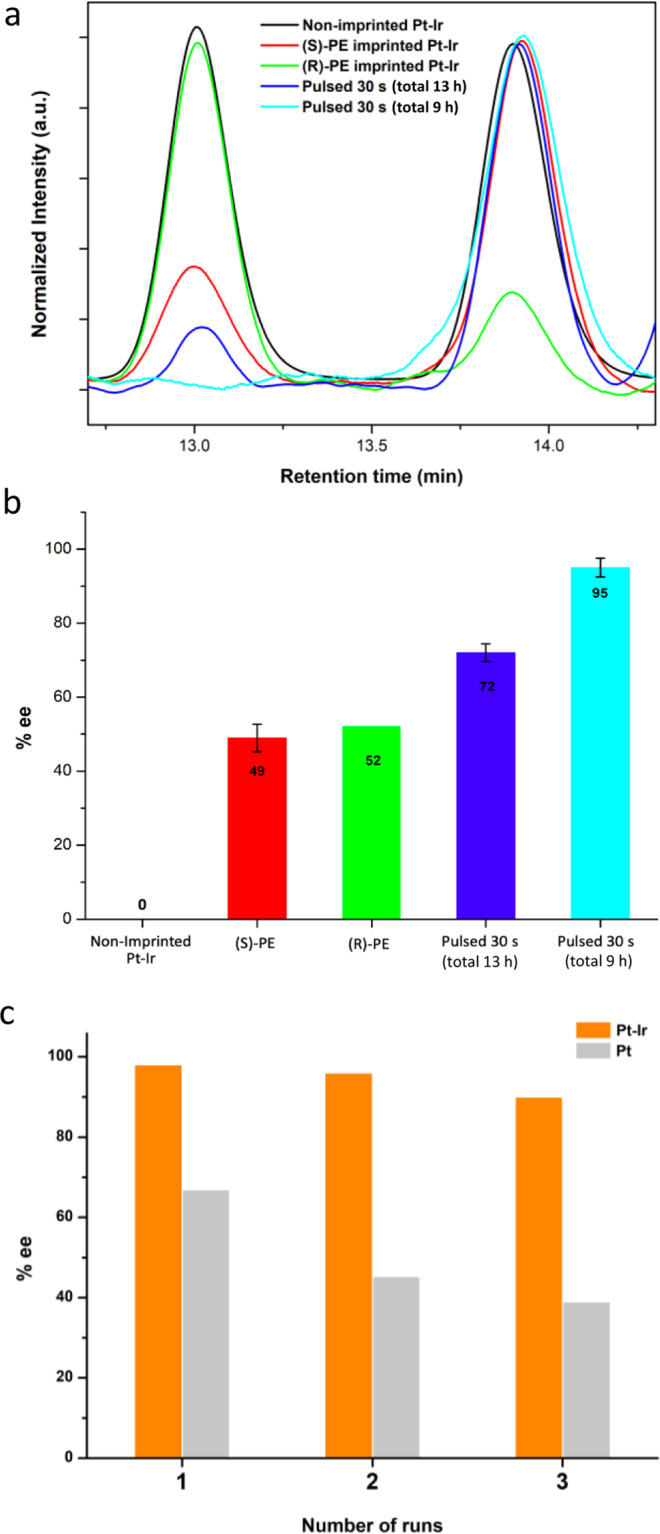
Enantioselective electrosynthesis. Analysis of reaction products after asymmetric electroreduction of acetophenone on chiral-imprinted Pt-Ir alloy surfaces. **a** HPLC chromatograms of chiral products obtained with potentiostatic electrosynthesis for 13 h and pulsed potential synthesis (pulse time 30 s) in 1 M NH_4_Cl (pH 4.0) at −0.35 V using the different Pt-Ir electrodes. **b** Histograms summarizing the enantiomeric excess (%ee) for non-imprinted Pt-Ir, (S)-PE (red), and (R)-PE (green) imprinted Pt-Ir electrodes, obtained by steady-state electrosynthesis, or (S)-PE-imprinted Pt-Ir electrodes used in pulsed electrosynthesis with a total pulse duration of 13 h (dark blue) and 9 h (light blue). **c** Comparative graph of the %ee obtained from reusability tests of a (S)-PE-imprinted Pt-Ir electrode (orange) and literature results obtained with a (S)-PE-imprinted monometallic Pt electrode^26^ (gray). Retention times of (R)-PE and (S)-PE are 13.0 and 13.9 min, respectively. The s.e.m. is 2.16 %ee.

As illustrated in the HPLC chromatograms (Fig. 5a) and histograms (Fig. 5b) of the electrosynthesis products, after a potentiostatic synthesis for 13 h, there is no enantioselectivity when using non-imprinted mesoporous Pt-Ir as a working electrode. However, when employing a chiral mesoporous Pt-Ir electrode encoded with (S)-PE, a remarkable enantiomeric excess of 49 ± 4% can be achieved under otherwise identical conditions. A mirror value of enantiomeric excess is obtained when using an electrode imprinted with (R)-PE (52%). The opposite sign of enantiomeric excess indicates that the chiral-imprinted Pt-Ir alloys are able to preferentially synthesize the corresponding enantiomers. Most importantly, the enantiomeric excess achieved with chiral-encoded Pt-Ir surfaces is much more pronounced compared to values reported previously for pure monometallic structures^26,27^. In order to further enhance the enantiomeric excess, a recently developed pulse electrosynthesis strategy was applied to suppress the competitive reactions at the non-imprinted sites^26,27^. In this case, the pulse duration and relaxation time is 30 s. Interestingly, the enantiomeric excess is very significantly improved to 72 ± 2% for the same global reaction time as for the steady-state electrosynthesis (13 h). The enantiomeric excess is even more dramatically enhanced to up to 95 ± 3% when decreasing the total duration of the pulses to 9 h. This might be due to the fact that for a longer global synthesis time, the above-mentioned by-product 2,3-diphenyl-2,3-butanediol is accumulating in the solution, as we could monitor by HPLC. As butanediol derivatives tend to adsorb on Pt^52–54^, this should lead to a gradual deactivation of the chiral surface spots when prolonging the synthesis time and thus to a less pronounced enantioselectivity.

Although a high enantiomeric excess of around 90% can be achieved with chiral-encoded monometallic Pt surfaces, as reported previously, this is only possible for very short potential pulses, and consequently a much longer total synthesis time is needed. When using the same pulse duration of 30 s with pure Pt electrodes, only 58 %ee could be obtained^26^, translating an almost two-fold increase in %ee for the alloy structures under analog experimental conditions. These observations confirm again the benefits of alloys which may be attributed to a more asymmetric environment in the bimetallic phase^33,49^, and lower adsorption energies of protons on the alloy in comparison with monometallic Pt and Ir. As has been shown by DFT calculations, the lower adsorption energy of protons on the alloy, compared to monometallic Pt and Ir, might facilitate the interaction between the prochiral molecules and the adsorbed protons, eventually leading to a higher electrochemical activity^44^. Moreover, the better stereoselectivity observed with Pt-Ir might be explained by the presence of more asymmetric steps or kink sites, due to the intersection of different crystal planes, playing a major role in the activity enhancements^12,38^. Pt-Ir deposits also have globally a smoother outer surface, thus decreasing the number of unspecific adsorption sites, and consequently increasing the enantioselectivity.

As already mentioned above, the stability of monometallic chiral electrodes is an important problem^26,27^. Hence, we carried out additional experiments to investigate the stability and reusability of the chiral-imprinted mesoporous alloys for several sequential electrosynthesis experiments. Supplementary Fig. 10 displays HPLC chromatograms of the reaction products obtained by pulsed electrosynthesis when using the same (S)-PE-imprinted mesoporous Pt-Ir electrode for three different experiments. The alloy is able to retain almost completely the chiral information, indicated by a decrease of the %ee by less than 10% (98 %ee in the 1^st^ run; 90 %ee in the 3^rd^ run). In strong contrast to this, a dramatic decrease in enantiomeric excess was recorded after three electrocatalytic cycles for chiral-imprinted monometallic Pt^26^ (66.9 %ee in the 1^st^ run; 39 %ee in the 3^rd^ run) (Fig. 5c). This again confirms the beneficial electrochemical stability of these nanostructured matrices, enabled by introducing just small amounts of Ir into the Pt bulk.

The slight decrease in enantioselectivity after several cycles can be explained by surface energy relaxation, inducing, eventually, segregation of metal atoms into nanoislands, and thus a gradual loss of activity^38,55^. Nevertheless, Pt-Ir is much more stable than Pt at the atomic scale, due to a more favorable local cohesive energy for the Ir sites (1.5 eV difference compared to Pt). This implies that when Pt-Ir is forming an alloy structure, the stability of the atomic arrangement is enhanced due to the influence of the inserted Ir atoms, compared to monometallic Pt^56^, providing a rational for the only very little degradation of enantioselectivity in the present study.

## Discussion

As illustrated in previous studies, the major obstacle for chiral-encoded mesoporous monometallic metals is the significantly decreasing enantioselectivity when using them for asymmetric synthesis in several electrocatalytic cycles. In order to overcome these drawbacks, it is necessary to improve the electrode material in terms of performance and durability. Alloying monometallic matrices with a small amount of a promoter is an appropriate strategy since the enhanced mechanical stability due to the different size of the metal atoms results in an improved hardness of the bulk structure. This positively affects the enantioselectivity and catalytic activity by changing the local surface energy.

In this contribution, Ir was selected as a promoter, forming an alloy with Pt, resulting in an increase of hardness for the bulk material and less surface reconstruction at the atomic level, as well as an impressive tolerance of drastic electrochemical conditions. Chiral-encoded mesoporous Pt-Ir alloys were successfully obtained by electroreduction of H_2_PtCl_6_ and H_2_IrCl_6_ mixtures in the simultaneous presence of a LLC and the desired chiral templates. Characterization by CV, SEM, and TEM reveals that the mesoporous Pt-Ir electrodes are composed of a well-ordered hexagonal pore structure and have a smooth external surface. By the addition of 10 at.% Ir into the Pt structure, both metals form an alloy where Ir is homogeneously dispersed in the Pt bulk, as confirmed by XRD and XPS results. The alloy structures are preferentially generated at the initial stages of electrodeposition and the Ir content is gradually decreasing in the upper layers as illustrated by XPS depth-profile analysis.

Different chiral molecules, such as DOPA and 1-PE, can be used as templates, and the chiral information is retained after removal of the templates. The nanoengineered alloy films exhibit significant features for enantioselective recognition and asymmetric synthesis of chiral compounds with an excellent mechanical and electrochemical stability compared to the monometallic analog. For example, (S)-PE-imprinted Pt-Ir films have a high activity and durability when used in enantioselective electroreduction, yielding almost 100 %ee during their first use, and still up to 90 %ee even after three subsequent electrosynthesis cycles. This corresponds to a three-fold increase in electrochemical stability compared to monometallic Pt electrodes. The report clearly illustrates the benefits of using alloys instead of pure metal phases in the frame of chiral recognition and enantioselective synthesis. The exceptional improvement of activity and durability of chiral-encoded metal alloys allows overcoming the current roadblocks for using these advanced materials as chiral sensors, enantiospecific adsorbers^57^, catalysts for asymmetric electrosynthesis, stereoselective heterogeneous catalysts^11^, and even as active materials in the frame of recently proposed strategies for asymmetric photocatalysis^58–60^. In addition to the intriguing fundamental aspects, this opens up very promising perspectives with respect to real-world applications.

## Methods

### Chemicals

Hexachloroplatinic acid hydrate (H_2_PtCl_6_·xH_2_O), hexachloroiridic acid hydrate (H_2_IrCl_6_·xH_2_O), polyethylene glycol hexadecyl ether (Brij^®^ C10), 3,4-dihydroxy-L-phenylalanine ((L)-DOPA) and 3,4-dihydroxy-D-phenylalanine ((D)-DOPA), (R)-phenylethanol, (S)-phenylethanol, acetophenone, isopropyl alcohol, heptane, and ammonium chloride (NH_4_Cl) were purchased from Sigma-Aldrich. Hydrochloric acid (HCl) and sulfuric acid (H_2_SO_4_) were obtained from Alfa. MilliQ water (18.2 MΩ·cm) was used for all experiments. All chemicals were directly used without further purification.

### Synthesis of mesoporous alloyed Pt-Ir electrodes

All electrochemical measurements were conducted using a potentiostat (Metrohm µAutolab Type III) equipped with a three-electrode system. Ag/AgCl (sat. KCl), a Pt mesh, and a cleaned gold-coated glass slide (0.25 cm^2^) were used as reference, counter, and working electrodes, respectively. Typically, a gold-coated glass slide was cleaned by isopropyl alcohol, washed with MilliQ water several times, and dried under N_2_ gas flow. The solution, composed of 29 wt% of metal precursors (mixing of H_2_PtCl_6_·6H_2_O and H_2_IrCl_6_·6H_2_O, with 85Pt:15Ir at.%), 29 wt% of MilliQ water and the desired amount of chiral template molecules was mixed by ultra-sonication for 5 min. Subsequently, 42 wt% of Brij^®^ C10 was added to the solution. The resulting paste was continuously mixed for 15 min and then heated at 40 °C for 45 min, repeating this cycle three times. The final LLC gel was positioned on the cleaned gold-coated glass slide. The electrodeposition process was carried out by chronoamperometry with a desired charge density at various deposition potentials. Finally, the electrode was soaked in MilliQ water overnight to remove the template molecules. The non-imprinted mesoporous Pt-Ir electrodes were synthesized following the above-mentioned process, without adding chiral templates to the LLC precursor gel.

### Characterization

The morphology of the Pt-Ir electrodes was characterized by SEM performed on a Hitachi TM-1000 tabletop microscope. Porosity and lattice fringes of Pt-Ir were investigated by TEM with a JEOL JEM-ARM200F microscope at 200 kV using ultra-thin Pt-Ir samples. Cross-sectional imaging and elemental analysis were performed by SEM equipped with EDS (SEM/EDS) on a JEOL, JSM-7610F. The elemental composition was measured by ICP-OES on an Agilent Technologies, 700 series. Prior to analysis, Pt-Ir samples were digested in aqua regia solution. In order to determine the Pt-Ir alloy structure, X-ray diffraction (XRD) patterns were recorded on a Bruker D8 ADVANCE instrument with CuKα radiation at 40 kV and 40 mA. To investigate the metallic state and the composition of the Pt-Ir films, XPS was performed on a JEOL JPS-9010 equipped with a monochromatic AlKα source with a low-energy electron flood gun for sample pretreatment. The C 1*s* peak at 284.7 eV was used as a reference to correct the positions of the spectra. For recording XPS depth profiles, the mesoporous Pt-Ir was etched by an argon ion gun for 160 s with an etching rate of approximately 2.67 nm s^−1^. Hence, the total thickness of the removed Pt-Ir layer after 160 s of etching is around 400 nm. Atomic percentages (at.%) of the compositions were calculated using the relative integrated peak areas of the Pt (Pt 4*f*_7/2_ and 4*f*_5/2_) and Ir (Ir 4*f*_7/2_ and Pt 4*f*_5/2_). CV was used to measure the ECSA of the prepared electrodes in 0.5 M H_2_SO_4_ at a scan rate of 100 mV s^−1^. All electrochemical experiments were carried out using a Metrohm µAutolab Type III equipment.

### Determination of chiral discrimination properties

Chiral recognition was investigated by using (L)- or (D)-DOPA-imprinted mesoporous Pt-Ir electrodes, obtained by using a deposition charge density of 6 C cm^−2^. The experiments were conducted by DPV (step potential 10 mV, modulation amplitude 50 mV, modulation time 50 ms, interval time 500 ms), with 4 mM DOPA in 50 mM HCl solution as supporting electrolyte (pH 1.4). To erase chiral information, the potential of the electrode was scanned in a full cycle in the range between −0.20 and 1.80 V by CV in 0.5 M H_2_SO_4_ solution.

### Enantioselective synthesis

Asymmetric electrosynthesis was tested by reduction of acetophenone to phenylethanol at potentials in the range from −0.4 to −0.3 V in a stirred solution (250 rpm) of 5 mM acetophenone in 1 M NH_4_Cl as supporting electrolyte. Different pH values from 5.0 to 2.0 were tested. After the reaction, the products were extracted by heptane and the solution was analyzed by HPLC carried out on a JASCO LC-Net II/ADC equipped with a chiral column (CHIRALPAK IB N-5, 250 × 4.6 mm inner diameter) and a photodiode array (PDA) detector at a wavelength of 210 nm. The analysis was performed at a flow rate of 0.5 ml min^−1^, using a mixture of 8/92 (v/v) isopropyl alcohol/heptane as mobile phase.

The enantiomeric excess (%ee) was calculated by using Eq. (1):

1 $${\mathrm{Enantiomeric}}\,{\mathrm{excess}}\,(\% {\mathrm{ee}}) = \left[ {\frac{{({\mathrm{R}}){\mathrm{PE}} - ({\mathrm{S}}){\mathrm{PE}}}}{{({\mathrm{R}}){\mathrm{PE}} + ({\mathrm{S}}){\mathrm{PE}}}}} \right] \times 100$$

where (R)PE and (S)PE are the integrated HPLC peak areas of (R)-PE and (S)-PE, respectively. The standard error of the mean of the enantiomeric excess (s.e.m.) is obtained by Eq. (2);

2 $${\mathrm{s}}.{\mathrm{e}}.{\mathrm{m}}. = \frac{S}{{\sqrt n }}$$

where *S* is the standard deviation and *n* the number of experiments (in the present case *n* = 3).

## Supplementary information

Supplementary information.

## Data Availability

The datasets generated and analyzed in the frame of the current study are available from the corresponding authors on request.

## References

[1] Bada JL (1995). Origins of homochirality. Nature.

[2] Zaera F (2017). Chirality in adsorption on solid surfaces. Chem. Soc. Rev..

[3] Panke S, Held M, Wubbolts M (2004). Trends and innovations in industrial biocatalysis for the production of fine chemicals. Curr. Opin. Biotechnol..

[4] Maier NM, Franco P, Lindner W (2001). Separation of enantiomers: needs, challenges, perspectives. J. Chromatogr. A.

[5] Pollock D, Waldvogel SR (2020). Electro-organic synthesis – a 21st century technique.. Chem. Sci..

[6] Bloom B (2020). Asymmetric reactions induced by electron spin polarization.. Phys. Chem. Chem. Phys..

[7] Noyori R (2002). Asymmetric catalysis: science and opportunities (nobel lecture). Angew. Chem. Int. Ed..

[8] Yoon TP, Jacobsen EN (2003). Privileged chiral catalysts. Science.

[9] Cornils B, Herrmann WA (2003). Concepts in homogeneous catalysis: the industrial view. J. Catal..

[10] Heitbaum M, Glorius F, Escher I (2006). Asymmetric heterogeneous catalysis. Angew. Chem. Int. Ed..

[11] Kyriakou G, Beaumont SK, Lambert RM (2011). Aspects of heterogeneous enantioselective catalysis by metals. Langmuir.

[12] Shukla N, Gellman AJ (2020). Chiral metal surfaces for enantioselective processes. Nat. Mater..

[13] Gellman AJ (2010). Chiral surfaces: accomplishments and challenges. ACS Nano.

[14] Riva S (2017). Chirality in metals: an asymmetrical journey among advanced functional materials. Mater. Sci. Technol..

[15] Wattanakit C (2018). Chiral metals as electrodes. Curr. Opin. Electrochem..

[16] Lorenzo MO (1999). Creating chiral surfaces for enantioselective heterogeneous catalysis: R, R-tartaric acid on Cu (110). J. Phys. Chem. B.

[17] Humblot V, Raval R (2005). Chiral metal surfaces from the adsorption of chiral and achiral molecules. Appl. Surf. Sci..

[18] Hazen RM, Sholl DS (2003). Chiral selection on inorganic crystalline surfaces. Nat. Mater..

[19] Attard GA (2001). Electrochemical studies of enantioselectivity at chiral metal surfaces. J. Phys. Chem. B.

[20] Yang L (2020). Chiral nanoparticles: chiral ligand‐free, optically active nanoparticles inherently composed of chiral lattices at the atomic scale. Small.

[21] Switzer JA, Kothari HM, Poizot P, Nakanishi S, Bohannan EW (2003). Enantiospecific electrodeposition of a chiral catalyst. Nature.

[22] Pachón LD (2009). Chiral imprinting of palladium with cinchona alkaloids. Nat. Chem..

[23] Wattanakit C (2014). Enantioselective recognition at mesoporous chiral metal surfaces. Nat. Commun..

[24] Yutthalekha T, Warakulwit C, Limtrakul J, Kuhn A (2015). Enantioselective recognition of DOPA by mesoporous platinum imprinted with mandelic acid. Electroanalysis.

[25] Yutthalekha T (2016). Asymmetric synthesis using chiral-encoded metal. Nat. Commun..

[26] Wattanakit C, Yutthalekha T, Asssavapanumat S, Lapeyre V, Kuhn A (2017). Pulsed electroconversion for highly selective enantiomer synthesis. Nat. Commun..

[27] Assavapanumat S, Ketkaew M, Kuhn A, Wattanakit C (2019). Synthesis, characterization, and electrochemical applications of chiral imprinted mesoporous Ni surfaces. J. Am. Chem. Soc..

[28] Wattanakit C, Kuhn A (2020). Encoding chiral molecular information in metal structures. Chem. Eur. J..

[29] Avnir D (2014). Molecularly doped metals. Acc. Chem. Res..

[30] Attard GS (1997). Mesoporous platinum films from lyotropic liquid crystalline phases. Science.

[31] Assavapanumat S (2019). Potential‐induced fine‐tuning of the enantioaffinity of chiral metal phases. Angew. Chem. Int. Ed..

[32] Assavapanumat S (2019). Chiral platinum–polypyrrole hybrid films as efficient enantioselective actuators. Chem. Comm..

[33] Elgavi H, Krekeler C, Berger R, Avnir D (2012). Chirality in copper nanoalloy clusters. J. Phys. Chem. C.

[34] Callister, W. D. & Rethwisch, D. G. *Materials Science and Engineering* Vol. 5 (John Wiley & Sons, 2011).

[35] Livingstone, S. E. *The Chemistry of Ruthenium, Rhodium, Palladium, Osmium, Iridium and Platinum: Pergamon Texts in Inorganic Chemistry* Vol. 25 (Elsevier, 2016).

[36] Rao CR, Trivedi D (2005). Chemical and electrochemical depositions of platinum group metals and their applications. Coord. Chem. Rev..

[37] Bu L (2015). A general method for multimetallic platinum alloy nanowires as highly active and stable oxygen reduction catalysts. Adv. Mater..

[38] Todoroki N, Watanabe H, Kondo T, Kaneko S, Wadayama T (2016). Highly enhanced oxygen reduction reaction activity and electrochemical stability of Pt/Ir(111) bimetallic surfaces. Electrochim. Acta.

[39] Kwon S (2018). Active methanol oxidation reaction by enhanced CO tolerance on bimetallic Pt/Ir electrocatalysts using electronic and bifunctional effects. ACS Appl. Mater. Interfaces.

[40] El Sawy EN, Birss VI (2013). A comparative study of the electrodeposition of nanoporous Ir and Pt thin films. J. Electrochem. Soc..

[41] Malgras V (2016). Nanoarchitectures for mesoporous metals. Adv. Mater..

[42] Sawy ENE, Molero HM, Birss VI (2014). Methanol oxidation at porous co-electrodeposited Pt-Ir thin films. Electrochim. Acta.

[43] Łukaszewski M, Soszko M, Czerwiński A (2016). Electrochemical methods of real surface area determination of noble metal electrodes–an overview. Int. J. Electrochem. Sci..

[44] Kwon S, Ham DJ, Lee SG (2015). Enhanced H_2_ dissociative phenomena of Pt–Ir electrocatalysts for PEMFCs: an integrated experimental and theoretical study. RSC Adv..

[45] Ramírez-Crescencio F (2017). Facile obtaining of iridium (0), platinum (0) and platinum (0)-iridium (0) alloy nanoparticles and the catalytic reduction of 4-nitrophenol. Mater. Chem. Phys..

[46] Ahmadi A, Attard G, Feliu J, Rodes A (1999). Surface reactivity at “chiral” platinum surfaces. Langmuir.

[47] Toda T, Igarashi H, Uchida H, Watanabe M (2019). Enhancement of the electroreduction of oxygen on Pt alloys with Fe, Ni, and Co. J. Electrochem. Soc..

[48] Stamenkovic VR (2007). Improved oxygen reduction activity on Pt_3_Ni(111) via increased surface site availability. Science.

[49] Prinz J, Gröning O, Brune H, Widmer R (2015). Highly enantioselective adsorption of small prochiral molecules on a chiral intermetallic compound. Angew. Chem. Int. Ed..

[50] Kodama Y, Imoto M, Ohta N, Kitani A, Ito S (2001). Selective reduction of acetophenone to 1-phenylethanol in aqueous media. J. Electroanal. Chem..

[51] Walczak MM, Dryer DA, Jacobson DD, Foss MG, Flynn NT (1997). pH Dependent redox couple: an illustration of the nernst equation. J. Chem. Educ..

[52] Gao P (1991). Studies of adsorbed saturated alcohols at platinum(111) electrodes by vibrational spectroscopy (EELS), auger spectroscopy, and electrochemistry. Langmuir.

[53] Hilmi A, Belgsir EM, Léger JM, Lamy C (1995). Electrocatalytic oxidation of aliphatic diols on platinum and gold part I: effects of chain length and isomeric position. J. Electroanal. Chem..

[54] Hazzazi OA, Attard GA, Wells PB (2004). Molecular recognition in adsorption and electro-oxidation at chiral platinum surfaces. J. Mol. Catal. A.

[55] Jacobse L, Rost MJ, Koper MTM (2019). Atomic-scale identification of the electrochemical roughening of platinum. ACS Cent. Sci..

[56] Dean J, Taylor MG, Mpourmpakis G (2019). Unfolding adsorption on metal nanoparticles: connecting stability with catalysis. Sci. Adv..

[57] Shukla N, Bartel MA, Gellman AJ (2010). Enantioselective separation on chiral Au nanoparticles. J. Am. Chem. Soc..

[58] Qiu X (2020). Applications of nanomaterials in asymmetric photocatalysis: recent progress, challenges, and opportunities.. Adv. Mater..

[59] Li S (2019). Single- and multi-component chiral supraparticles as modular enantioselective catalysts. Nat. Commun..

[60] Liang C, Huang J, Zhang X (2016). Effects of engineered nanoparticles on the enantioselective transformation of metalaxyl agent and commercial metalaxyl in agricultural soils. J. Agric. Food Chem..

