# Supplementary material for: Nanoengineered chiral Pt-Ir alloys for high-performance enantioselective electrosynthesis
Source: Nat Commun. 2021 Feb 26;12:1314. doi: 10.1038/s41467-021-21603-8 (PMC7910542; doi:10.1038/s41467-021-21603-8)
Supplement: Supplementary file 1 — Supplementary information. [file 41467_2021_21603_MOESM1_ESM.pdf]

Supplementary Information

Nanoengineered Chiral Pt-Ir Alloys for High-Performance Enantioselective Electrosynthesis

Sopon Butcha,[1,2] Sunpet Assavapanumat, [2] Somlak Ittisanronnachai, [2] Veronique Lapeyre,[1] Chularat Wattanakit,[2]* and Alexander Kuhn[1,2]*

[1] University of Bordeaux, CNRS UMR 5255, Bordeaux INP, Site ENSCBP, 16 avenue Pey Berland, 33607, Pessac, France.

[2] School of Molecular Science and Engineering and School of Energy Science and Engineering, Vidyasirimedhi Institute of Science and Technology, 21210, Rayong, Thailand.

*Corresponding authors e-mail: chularat.w@vistec.ac.th (C.W); kuhn@enscbp.fr (A.K.)

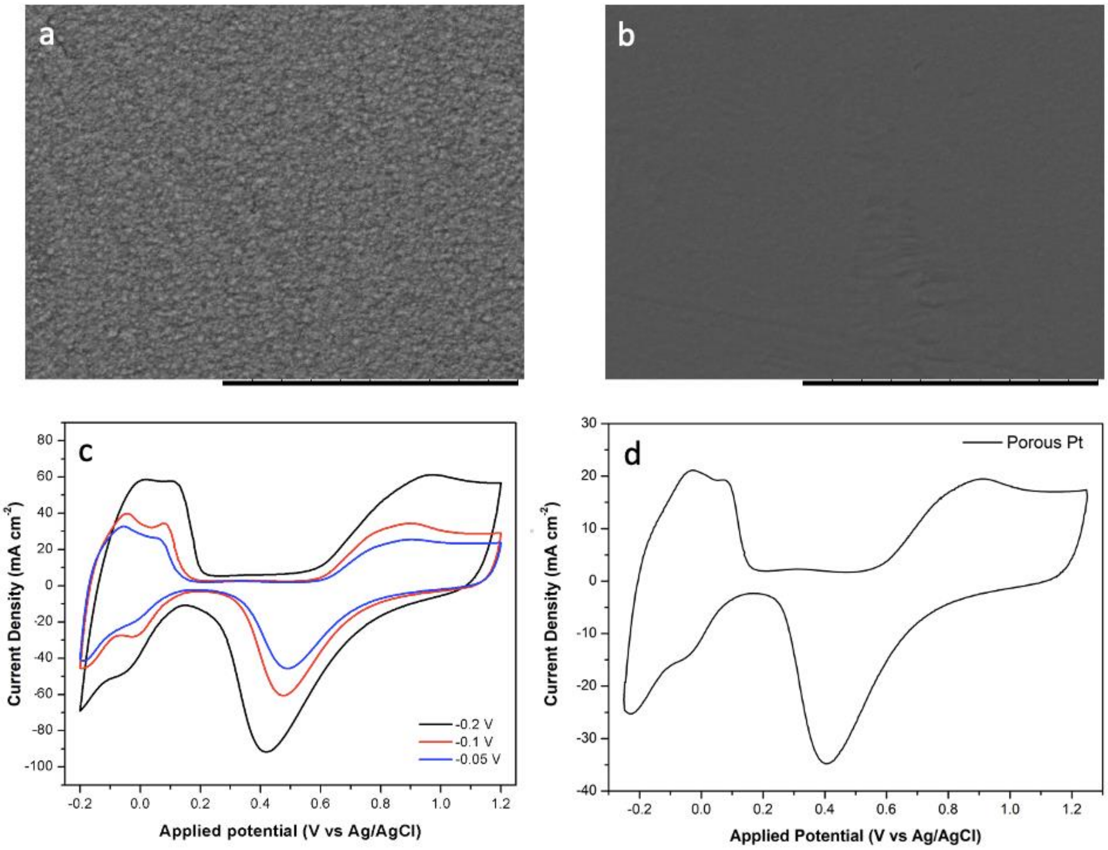

Supplementary Fig. 1: Characterization of mesoporous Pt-Ir films

a and **b** SEM images of mesoporous Pt-Ir electrodes deposited at -0.2 and -0.10 V, respectively, with a deposition charge density of 10 C cm^{-2} (scale bar 10 μm). **c** Cyclic voltammograms (CV) of mesoporous Pt-Ir electrodes synthesized with different electrodeposition potentials with a deposition charge density of 10 C cm^{-2}. **d** CV of a mesoporous monometallic Pt electrode with a deposition charge density of 8 C cm^{-2}. All CVs were recorded in 0.5 M H_2SO_4 at a scan rate of 100 mV s^{-1}.

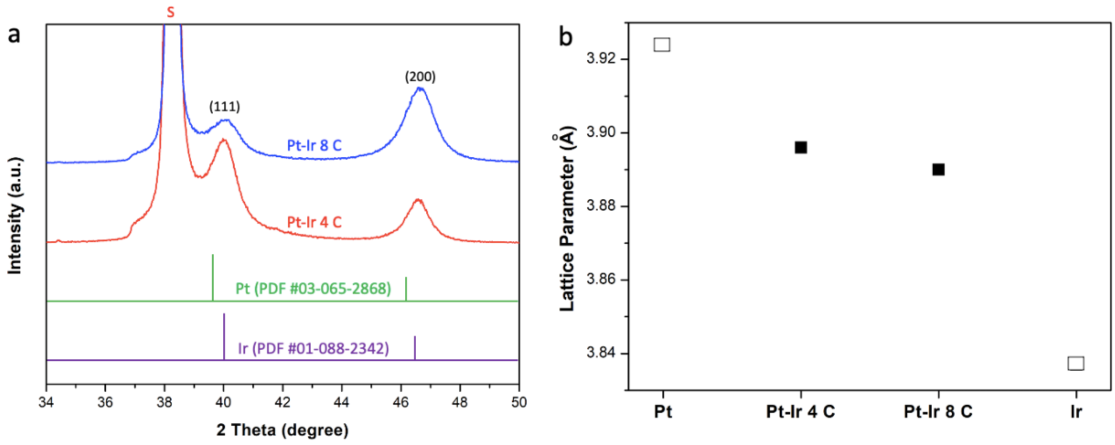

Supplementary Fig. 2: XRD analysis

a XRD pattern of the deposited Pt-Ir alloys with different thickness corresponding to deposition charge densities of 4 C cm^{-2} (red) and 8 C cm^{-2} (blue), respectively. Reference data of standard Ir (purple) and Pt (green) fcc structures (Pt, PDF #03-065-2868; Ir, PDF #01-088-2342) are included for comparison. The signal of a gold-coated glass slide used as a substrate (labelled with “s”) is observed at 38 degree. **b** Relationship between lattice parameters of the pure metals and the Pt–Ir alloy samples with different thicknesses. The reference lattice parameters for Pt and Ir are taken from Ref. [1].

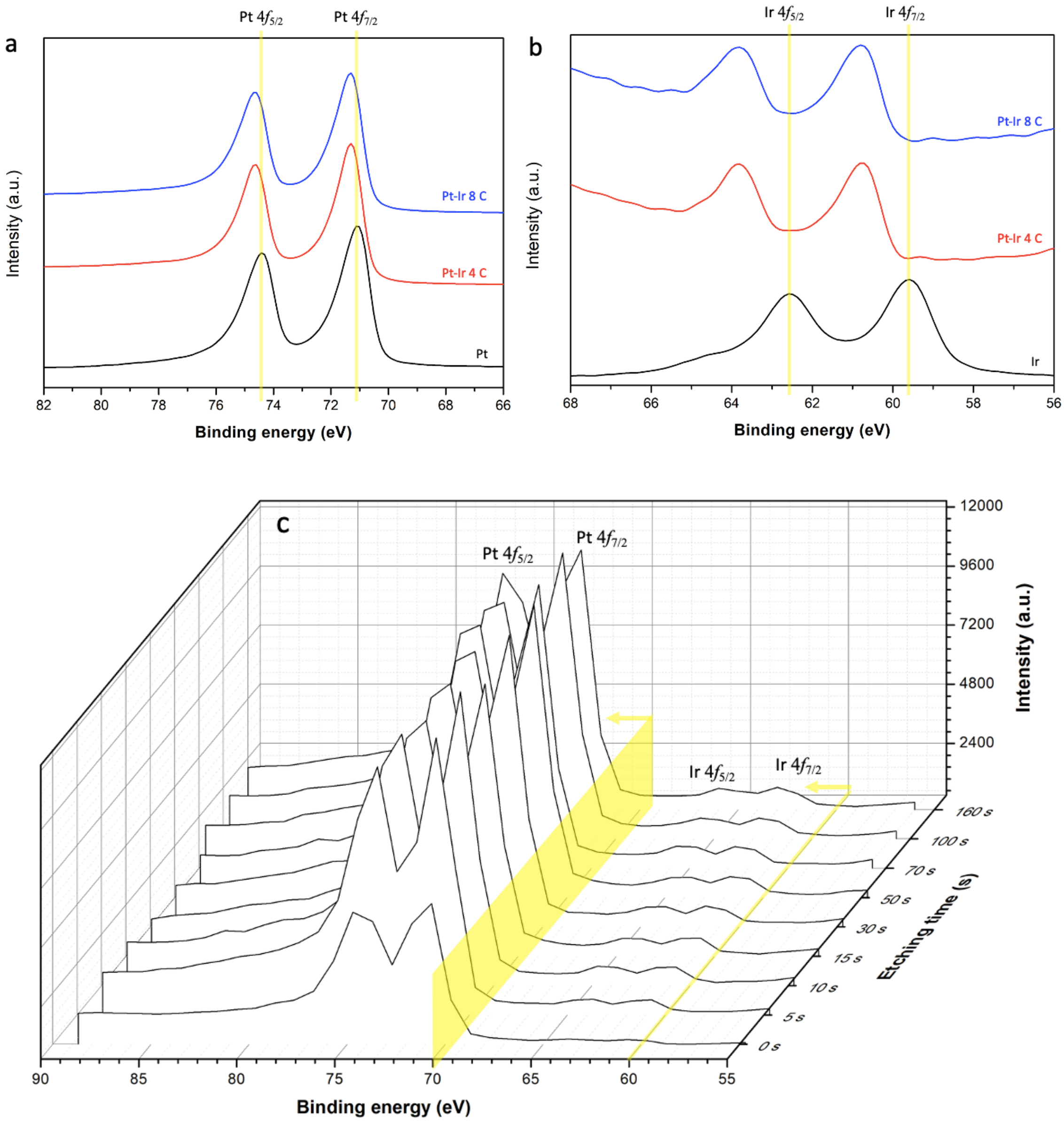

Supplementary Fig. 3: XPS spectra of the electrodes

a XPS spectra of the Pt standard (black) as well as for Pt-Ir 4 C (red) and 8 C (blue) in the binding energy (BE) region of Pt. **b** XPS spectra of the Ir standard (black) as well as for Pt-Ir 4 C (red) and Pt-Ir 8 C (blue) in the BE region of Ir. **c** Depth-profile XPS spectra of the alloy Pt-Ir 8 C in the binding energy (BE) regions of Pt and Ir as a function of etching time ranging from 0 to 160 s. The yellow plane and line indicate the initial onset of the lower energy peak for Pt and Ir, respectively, before etching.

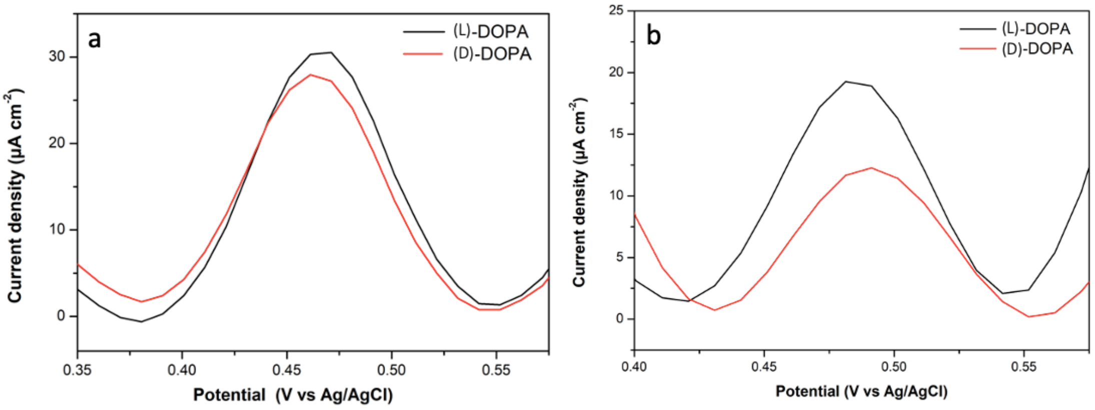

Supplementary Fig. 4: Selectivity comparison
Differential pulse voltammograms (DPVs) of the electrooxidation of 4 mM (L)-DOPA (black) and (D)-DOPA (red) in 50 mM HCl solution with different electrodes. **a** Non-imprinted mesoporous Pt-Ir. **b** Fresh (L)-DOPA imprinted mesoporous monometallic Pt.

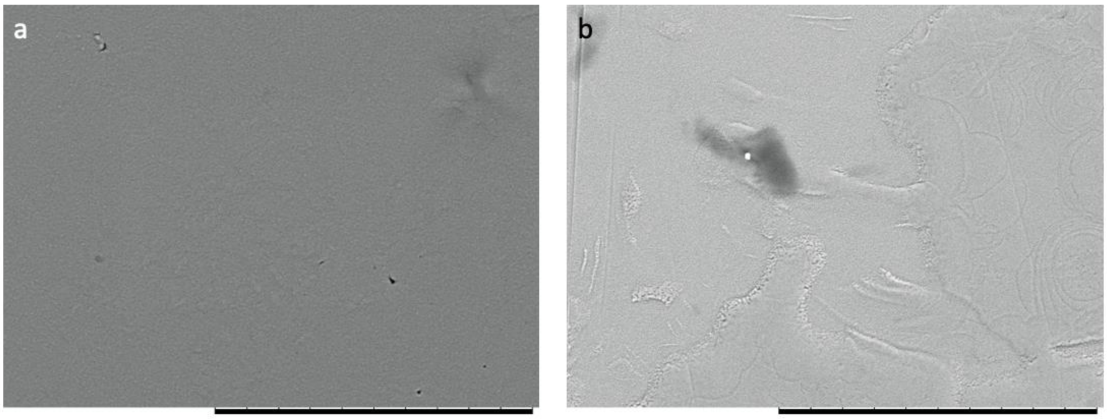

Supplementary Fig. 5: Surface morphology characterization
SEM images of porous metal electrodes obtained by electrodeposition with a deposition charge density of 6 C cm^{-2}. **a** Porous Pt-Ir alloy. **b** Porous monometallic Pt electrode (scale bars of 20 µm).

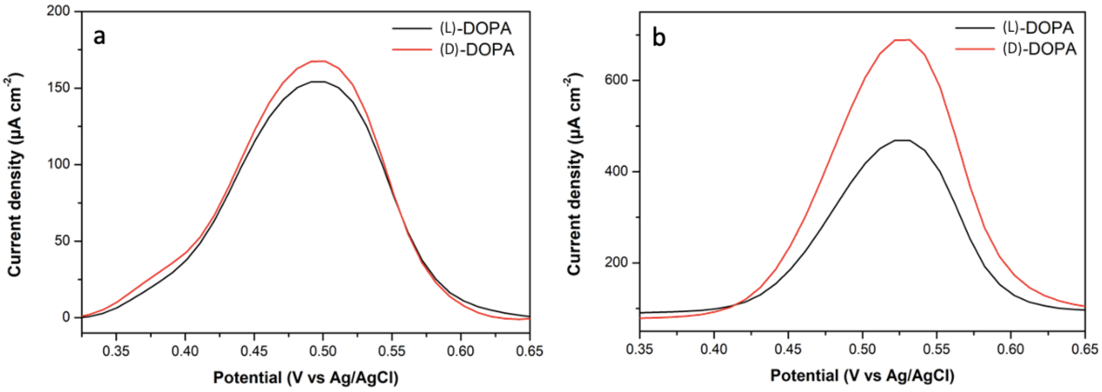

Supplementary Fig. 6: Stability of enantioselective detection

Differential pulse voltammograms (DPVs) of the electrooxidation of 4 mM (L)-DOPA (black) and (D)-DOPA (red) in 50 mM HCl solution for different electrodes. **a** (L)- DOPA imprinted mesoporous monometallic Pt. **b** (D)- DOPA imprinted mesoporous Pt-Ir after cycling the potential of both electrodes ten times in the range from -0.20 to 1.20 V in 0.5 M H_2SO_4.

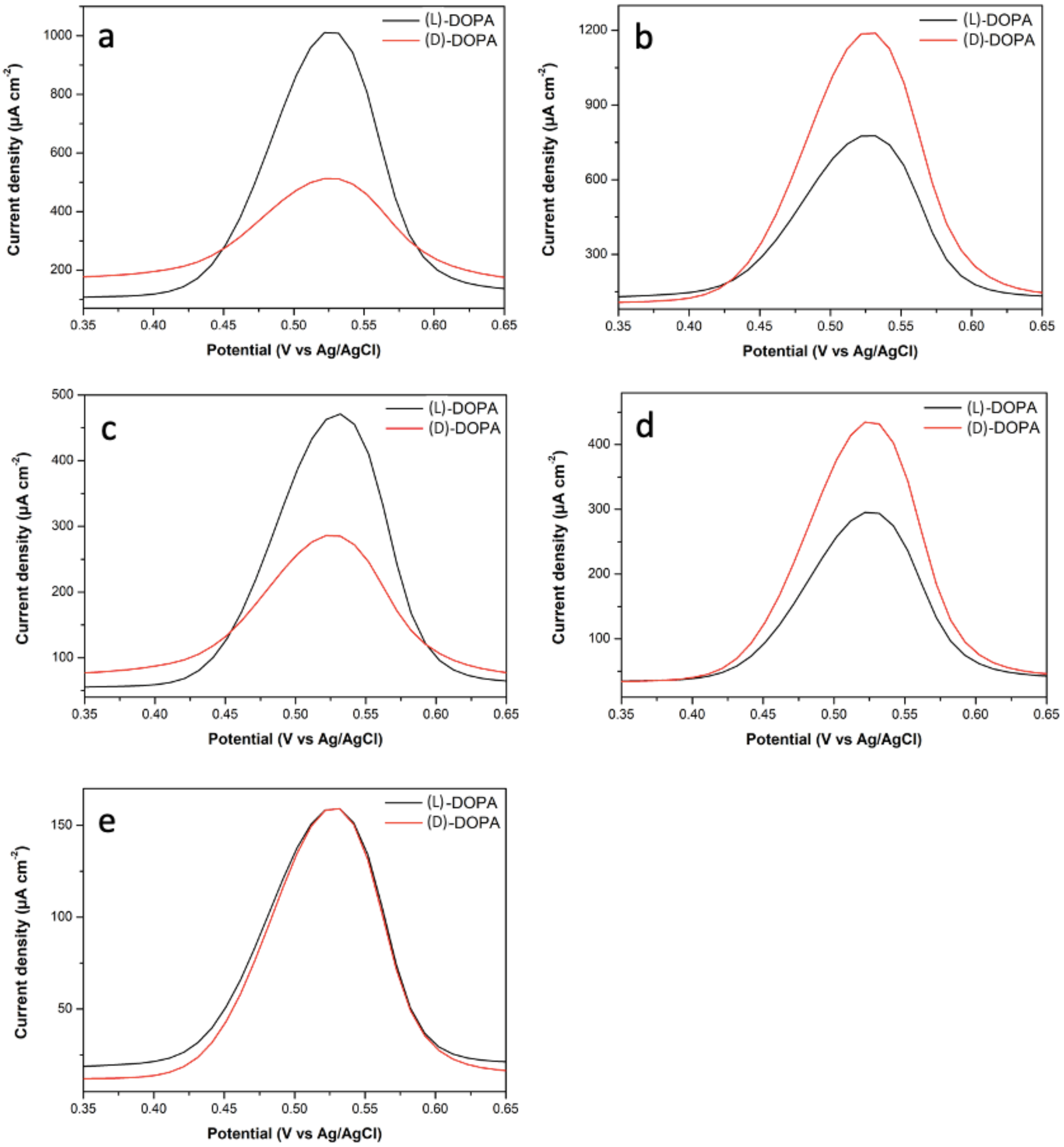

Supplementary Fig. 7: Stability of enantioselectivity under harsh conditions
Differential pulse voltammograms (DPVs) of the electrooxidation of 4 mM (L)-DOPA (black) and (D)-DOPA (red) in 50 mM HCl solution for different Pt-Ir electrodes. **a** and **b** (L)- DOPA and (D)- DOPA imprinted mesoporous Pt-Ir after running 20 cycles from -0.20 to 1.20 V. **c** and **d** (L)- DOPA and (D)- DOPA imprinted mesoporous Pt-Ir after erasing chiral information for 30 cycles from -0.20 to 1.40 V; **e** (*D*)- DOPA imprinted mesoporous Pt-Ir after erasing chiral features for 40 cycles from -0.20 to 1.80 V. All cycling experiments for erasing chiral information were performed by CV in 0.5 M H_2SO_4.

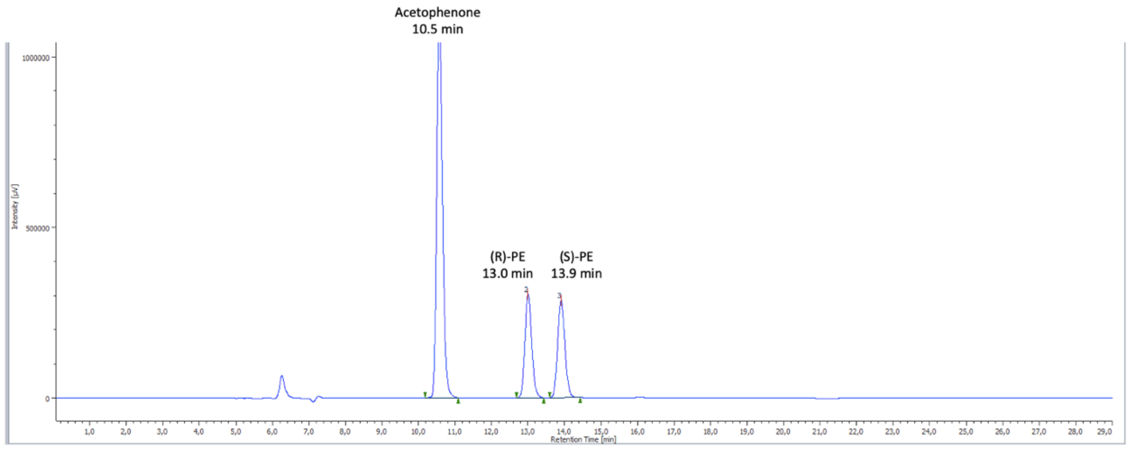

Supplementary Fig. 8: HPLC characterisation of the reaction products
Product solution after electroreduction of acetophenone using a non-imprinted porous Pt-Ir alloy as working electrode. Retention times of acetophenone, (R)-PE and (S)-PE are 10.5, 13.0 and 13.9 min, respectively. The reaction was performed by pulsed electrosynthesis with a pulse and relaxation time of 20 and 60 s, respectively. The conversion of acetophenone to PE is 39%.

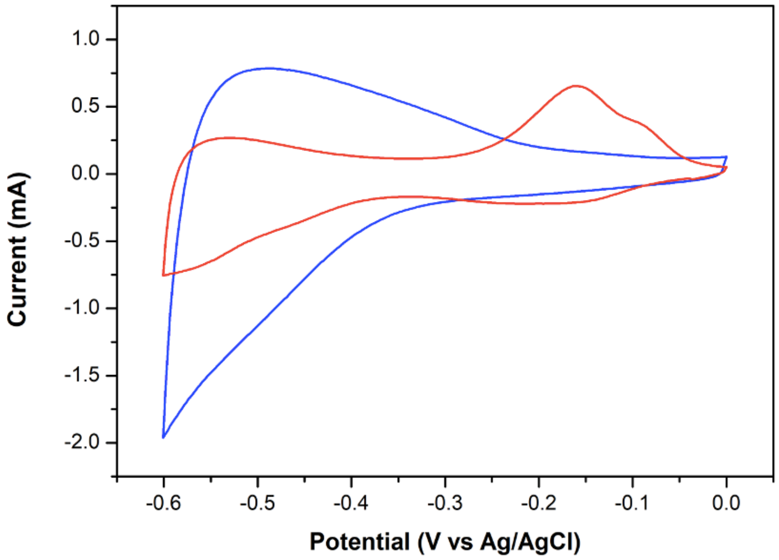

Supplementary Fig. 9: Determination of electroreduction onset potential
Cyclic voltammograms (scan rate of 100 mV/s) of a mesoporous Pt-Ir electrode in 1 M NH_4Cl (red) and in a solution containing also 40 mM acetophenone (blue), pH 5.0. The onset potential for the reduction of acetophenone is in the range from -0.3 to -0.4 V.

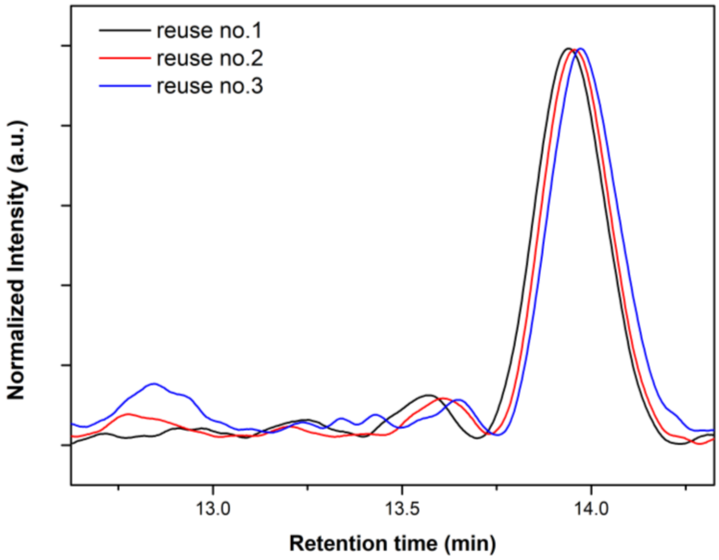

Supplementary Fig. 10: Selectivity stability for chiral electrosynthesis
HPLC chromatograms of product solution obtained from pulsed electrosynthesis when reusing the same (S)-PE imprinted Pt-Ir electrode for three subsequent experiments.

Supplementary Table 1: Optimization of electrodeposition conditions
Effect of electrodeposition potential and deposition charge density on the morphology and elemental composition of mesoporous Pt-Ir electrodes. Pt and Ir at% values are estimated from EDS-SEM measurements. The roughness factor is calculated by dividing the electrochemically active surface by the geometrical area (0.25 cm^{-2} in all cases) of the electrode. The roughness factor of the mesoporous Pt electrode of Supplementary Fig. 1d is also calculated to compare with mesoporous Pt-Ir obtained with the same deposition charge density of 8 C cm^{-2}. The values for Pt and Pt-Ir are 324 and 400, respectively.

Applied potential (V)	Charge density (C cm^{-2})	Real surface areas (S_r, cm^2)	Roughness factor	Pt (at%)	Ir (at%)
-0.20	10	649	2,596	100.0	-
-0.10	10	384	1,536	100.0	-
-0.05	10	354	1,416	99.5	0.5
-0.05	8	100	400	97.5	2.5
-0.05	6	66	264	97.0	3.0

Supplementary Table 2: Elemental analysis

As EDS is not perfectly well-suited for quantitative elemental analysis, due to a detection which is limited to selected and outermost areas of a sample, further quantitative analysis of the Pt-Ir samples was carried out with XPS and ICP-OES.

Samples	Elements	Theoretical at%	at% from EDS	at% from XPS	at% from ICP-OES
Pt-Ir 4 C	Pt	85.00	95.0	89.0	90.5
	Ir	15.00	5.0	11.0	9.5
Pt-Ir 8 C	Pt	85.00	97.5	91.5	90.7
	Ir	15.00	2.5	8.5	9.3

Supplementary Table 3: Stability characterisation during electrosynthesis

Rounded relative electrooxidation peak areas of the different chiral imprinted electrodes extracted from DPVs. Peak areas of either (L)-DOPA or (D)-DOPA signals were calculated with OriginPro version 8.5. A_L/A_D is determined for (L)-DOPA imprinted electrodes, while A_D/A_L is calculated for (D)-DOPA imprinted electrodes.

Electrodes	Peak Area of (L)-DOPA (A_L)	Peak Area of (D)-DOPA (A_D)	Relative peak area	
			A_L/A_D	A_D/A_L
(L)-DOPA Pt	0.0003	0.0002	1.5	-
Erased (L)-DOPA Pt after 10 cycles	0.0486	0.0518	0.9	-
(L)-DOPA Pt-Ir	0.0246	0.0017	14.5	-
Erased (L)-DOPA Pt -Ir after 10 cycles	0.2409	0.1817	1.3	-
Erased (L)-DOPA Pt -Ir after 20 cycles	0.2853	0.2173	1.3	-
Erased (L)-DOPA Pt -Ir after 30 cycles	0.1355	0.1088	1.2	-
Erased (L)-DOPA Pt -Ir after 40 cycles	0.0993	0.0982	1.0	-
(D)-DOPA Pt-Ir	0.0021	0.0284	-	13.5
Erased (D)-DOPA Pt -Ir after 10 cycles	0.1578	0.2129	-	1.4
Erased (D)-DOPA Pt -Ir after 20 cycles	0.2479	0.3404	-	1.4
Erased (D)-DOPA Pt -Ir after 30 cycles	0.0865	0.1190	-	1.3
Erased (D)-DOPA Pt -Ir after 40 cycles	0.0466	0.0432	-	0.9

Supplementary Table 4: pH studies
Effect of pH on product selectivity for the electroreduction of acetophenone with a (S)-PE imprinted Pt-Ir electrode.

pH of reactant solution	Applied Potentials (V vs. Ag/AgCl)	%Enantiomeric excess of (S)-PE
5.0	-0.40	28
4.0	-0.35	49
3.0	-0.30	24
2.0	-0.30	2

Supplementary References

[1] Wu, F. *et al*. Electrodeposition of Pt–Ir alloys on nickel-base single crystal superalloy TMS-75. *Surf. Coat. Technol*. **184**, 24–30 (2004).
